# Implementation of a diabetes prevention program within two community sites: a qualitative assessment

**DOI:** 10.1186/s43058-022-00258-6

**Published:** 2022-02-05

**Authors:** Tineke E. Dineen, Corliss Bean, Mary E. Jung

**Affiliations:** 1 School of Health and Exercise Sciences, University of British Columbia, Okanagan Campus, 3333 University Way, Kelowna, BC V1V 1V7 Canada; 2 Department of Recreational and Leisure Studies, Brock University, 1812 Sir Isaac Brock Way, St Catharines, ON L2S 3A1 Canada

**Keywords:** Implementation evaluation, Implementation science, Health behavior, Prediabetic state, Diet, Exercise

## Abstract

**Background:**

Despite numerous translations of diabetes prevention programs, implementation evaluations are rarely conducted. The purpose of this study was to examine the implementation process and multilevel contextual factors as an evidence-based diabetes prevention program was implemented into two local community organization sites to inform future scale-up. To build the science of implementation, context and strategies must be identified and explored to understand their impact.

**Methods:**

The program was a brief-counseling diet and exercise modification program for individuals at risk of developing type 2 diabetes. A 1-year collaborative planning process with a local not-for-profit community organization co-developed an implementation plan to translate the program. A pragmatic epistemology guided this research. Semi-structured interviews were conducted with staff who delivered the program (*n* = 8), and a focus group was completed with implementation support staff (*n* = 5) at both community sites. Interviews were transcribed verbatim and thematically analyzed using a template approach. The consolidated framework for implementation research (CFIR) is a well-researched multilevel implementation determinant framework and was used to guide the analysis of this study. Within the template approach, salient themes were first inductively identified, then identified themes were deductively linked to CFIR constructs.

**Results:**

Implementation strategies used were appropriate, well-received, and promoted effective implementation. The implementation plan had an impact on multiple levels as several CFIR constructs were identified from all five domains of the framework: (a) process, (b) intervention characteristics, (c) outer setting, (d) inner setting, and (e) individual characteristics. Specifically, results revealed the collaborative 1-year planning process, program components and structure, level of support, and synergy between program and context were important factors in the implementation.

**Conclusion:**

This study offers insights into the process of implementing a community-based diabetes prevention program in two local sites. Successful implementation benefited from a fully engaged, partnered approach to planning, and subsequently executing, an implementation effort. The CFIR was a useful and thorough framework to evaluate and identify multilevel contextual factors impacting implementation. Results can be used to inform future implementation and scale-up efforts.

**Supplementary Information:**

The online version contains supplementary material available at 10.1186/s43058-022-00258-6.

## Contributions to the literature

- Comprehensive reporting of implementation strategies, context, and processes that contribute to implementation are needed to advance the field and support future implementation and scale-up studies.
- Despite numerous translations of diabetes prevention programs, implementation evaluations describing the implementation process are rarely conducted and are needed to help advance the field.
- The Consolidated Framework for Implementation Research was a useful and thorough framework to guide data analysis
- As diabetes continues to rise, diabetes prevention programs are needed to meet the rising need. Fitness centers may be feasible community-based venues to deliver a sustainable individual, brief-counseling diabetes prevention program

## Background

Worldwide, 837 million people are diagnosed with, or at risk for, type two diabetes (T2D), costing 10% of global health expenditure [1]. Individuals with T2D have increased risk for kidney disease, cardiovascular disease, eye disease, and foot and lower limb complications [1]. With the economic and health burdens of T2D, increased attention is needed to prevent diabetes. Diet and exercise modification programs have demonstrated success in reducing future risk of developing T2D by up to 80% [2, 3] and indicated greater long-term effectiveness than pharmaceutical interventions (5.7–9.4 years) [4]. However, these efficacy studies have been executed under intensive, tightly controlled experimental conditions and are not suitable to implement and sustain in real life settings [5]. To reach the large number of at-risk individuals, programs must be implemented in community settings in a sustainable and scalable way.

To date, there have been multiple translations of diabetes prevention programs (e.g., [6–8]) with demonstrated effectiveness (e.g., [9, 10]). Despite many countries developing large-scale translation efforts (e.g., the USA, India, Australia, see [11]), there has been a lack of reporting on the implementation process, strategies, and context. Further, there have been no large-scale translations within a Canadian context. Implementation, as defined by Greenhalg, is the “active and planned efforts to mainstream an innovation within an organization” ([12] p582). There is a dearth of research in this area. A meta-narrative review of 495 sources on diffusion of innovations in healthcare identified the most serious research gap as limited research examining the processes of implementing and maintaining innovation in health service delivery and organizations in specific contexts [12]. Understanding the process and strategies used across studies can improve the field of implementation science by identifying effective strategies to improve future implementation efforts.

The largest translation study to date, the U.S. National Diabetes Prevention Program, conducted an evaluation aimed to report from an implementation perspective [13]. Successful scale-up was demonstrated, and promising site-level and program-level features were documented to boost engagement [13]. However, this study did not provide in-depth information on how or why such features were implemented, lacked description of the scale-up process, did not include provider-level perspectives, and did not describe specific implementation strategies. Such information is essential for understanding mechanisms leading to successful implementation. The Kerala Diabetes Prevention Program is a translational study that was culturally adapted to Kerala, India [14], and a thorough implementation evaluation has been conducted [15]. The evaluation of the cultural adaptation reported several contextual details including the impact of community stakeholders as a success factor and the use of a local resource person. In addition, the implementation evaluation reported several important implementation markers (e.g., feasibility, acceptability) from multiple perspectives. However, the study did not report specific implementation strategies. The present research builds on previous research by conducting a process evaluation of implementing a diabetes prevention program within a community organization by specifying the implementation process, strategies, and context.

Greenhalg identified context and confounders as a paradox at the center of implementation efforts that must be studied to understand what leads to success or lack of success of an implementation effort [12]. For example, within the National Diabetes Prevention Program, only 25% of sites received full Centre for Disease Control recognition within the study period (implemented the program for 12 months and achieved program outcomes per the Standards and Operating Procedures, necessary to continue to receive funding) [13]. To increase the likelihood of future success, in-depth evaluations on implementation processes can help identify contextual features from sites where there was successful, or unsuccessful, implementation. Building on the work of Greenhalg, and 18 other theories, the Consolidated Framework for Implementation Research (CFIR) was developed as a multilevel framework to systematically assess the implementation context to understand, “what works, where, and why” ([16] p2). The CFIR framework established a list of factors likely to influence implementation through individual, program, and organizational levels resulting in 39 fully operationalized constructs organized into five domains (intervention, inner setting, outer setting, individuals, and process) [16]. The intervention domain includes eight constructs associated with characteristics of the intervention that may influence implementation (e.g., complexity). The inner setting domain includes 12 constructs associated with characteristics of the organization that is implementing the intervention (e.g., culture). The outer setting domain includes four constructs associated with characteristics of the broader environment in which the organization is situated (e.g., cosmopolitanism). The individual domain includes five constructs associated with characteristics of individuals involved with the implementation (e.g., self-efficacy). Finally, the process domain includes eight constructs associated with strategies used to implement the intervention within the organization (e.g., opinion leader). In the current study, CFIR was used to systematically identify relevant factors that contribute to the success (or lack of success) of implementation, at multiple levels of the implementation context.

The CFIR has been adopted and used widely. In 2015, a systematic review identified 429 unique articles referencing the framework; however, the authors, including the author of the CFIR framework, stated only 6% of studies used CFIR in a meaningful manner [17]. Meaningful was defined as “to guide data collection, measurement, coding, analysis, and/or reporting” ([17] p2). The review also provided recommendations to help researchers apply the CFIR. This current study applied two of the reviews’ recommendations through (1) justifying selection of CFIR constructs (see the “Methods” section) and (2) meaningfully using the CFIR across different phases of implementation. In this present study, meaningful use of the CFIR occurred by using a during-implementation evaluation to inform future program scale-up initiatives. To date, the CFIR has been used in a pre-implementation study for integrating community health workers in primary care for diabetes prevention [18], in a protocol evaluating the implementation of a Veterans Affairs diabetes prevention program [19] and in community-based diabetes self-management education classes [20]. To our knowledge, this research is the first to use the CFIR to examine a community-based diabetes prevention program.

The present study utilized a qualitative approach to evaluate the process of implementing a diabetes prevention program within two sites of a local community organization. The purpose of this research was to examine the multilevel factors that facilitated or impeded implementation to inform future scale-up. Understanding the process of implementation, as well as the key contextual factors as the program enters a new organizational context, will help explain the success (or lack of success) of implementation and provide useful information to inform future scale-up. CFIR represents an ideal fit to help elucidate important process factors as it is a comprehensive, multi-level implementation determinant framework with strong theoretical heritage and fully operationalized constructs. This study contributes to the field a thorough implementation evaluation, allowing for future studies to understand the implementation process, strategies and factors impacting implementation.

## Methods

### Program description and study setting

Small Steps for Big Changes (SSBC) is an evidenced-based diabetes prevention program with demonstrated success on health-related outcomes (e.g., improved cardiorespiratory fitness, moderate to vigorous physical activity) [21–25]. The program uses a client-centered approach to support adults (age 18+) at risk of developing T2D make diet and exercise modifications. The 3-week program set in Kelowna, British Columbia, includes six one-on-one sessions of brief-counseling and supervised exercise. The program has been previously described (see [26] and supplementary file A for full program details). Working with the community partner, a local not-for-profit community organization (YMCA of Okanagan)^1^ housed within a broader national organization, a 1-year planning process facilitated co-development of a plan to sustainably translate the program into local community sites, with a goal to scale-up in the future [27]. The planning process engaged multiple stakeholders in face-to-face meetings, including a planning task force (consisting of researchers and upper-level YMCA leadership), site managers, prospective staff from the YMCA to enroll in the project, program alumni, and an external facilitator who guided two planning meetings (see [27] for further details). Throughout this process, both partners emphasized the importance of program fidelity, sufficient training, ongoing support, and open communication. These key factors were then targeted through the development of implementation strategies. In response to multiple calls for consistent language in the implementation field [16, 28], the strategies have been identified and described using the comprehensive list and definitions by Powell and colleagues (see Table 1) [28]. In doing so, this study will contribute to building an implementation evidence-base to support future implementation studies. The implementation plan was then executed to examine if SSBC can be successfully implemented within, and delivered by, YMCA staff. This current study examined the multilevel factors that facilitated or impeded the implementation process.

**Table 1:** Identification and description of implementation strategies used in SSBC

Implementation strategies	Description of strategy in SSBC
Develop and implement tools for quality monitoring	Developed session-specific fidelity checklists for staff to complete after every session. Data is collected, inputted, and evaluated for fidelity purposes by research team. Staff are instructed to audio record every session to be reviewed by study staff for motivational interviewing fidelity and program fidelity.
Audit and feedback	Staff audio-recorded every session and research staff reviewed randomly selected sessions to assess for motivational interviewing fidelity and program fidelity. To assess program fidelity, after each session staff completed session-specific checklists documenting what was delivered to their client. Provided feedback through evaluation reports.
Centralize technical assistance	A project coordinator dedicated to assisting the implementation efforts. All staff, site leads, and site managers had the contact information for the project coordinator.
Organize clinician implementation team meetings	Project coordinator planned and led mandatory 1-h monthly site meetings with all program staff.
Create a learning collaborative	Project coordinator planned and led monthly team meetings with staff involved in the project. Project coordinator provided a skeleton framework of support and encouraged staff to seek support from each other beyond site meetings.
Capture and share local knowledge	Local knowledge from implementation sites was shared through the project coordinator from site to site. In addition, this local knowledge was shared at the implementation team meetings. This sharing of information generated interest and led to the confirmation of the third site in the local area, as well as with the region and beyond.
Build a coalition	During the planning process an implementation team formed. This team consisted of three stakeholders from the YMCA and three stakeholders from the research team committed to the project. The team met monthly.
Use advisory boards and workgroups	Monthly implementation team meetings discussed implementation progress, solved challenges that arose, developed marketing and communication plans and continued to plan future scale-up strategies. A client advisory committee provided feedback on intervention content and tools (e.g., recruitment letter, client workbook) A stakeholder advisory committee provided feedback on program promotion, recruitment, and marketing.
Tailor strategies	Continuous feedback was collected from site leads, monthly meetings, and individual interviews with staff after 3 clients. Feedback was collected and used to tailor implementation strategies (e.g., training program, additions to training manual)
Purposely re-examine the implementation	Implementation strategies were reviewed at monthly site meetings and implementation team meetings, minor adjustments were made (e.g., adaptations to the training based on staff feedback).
Conduct educational meetings	Two local community events were held with multiple stakeholder groups attending (e.g., physicians, local health authority staff, university staff, community members, YMCA staff). Goals of these meetings were to provide updates to the community on study progress, distribute results, share success stories, and inform the community on the overall initiative to support stakeholder involvement, recruitment and support.
Conduct educational outreach visits	Research staff met with local physicians to educate practitioners about the program, promote recruitment and review process for referring eligible patients.
Develop a formal implementation blueprint	An implementation plan was created to support the program including standard operating procedures, a document describing the short- and long-term goals, roles and responsibilities, timeline, outcomes, and strategies for the project. The document was reviewed iteratively by both partners.
Develop academic partnerships	In 2017, the research team partnered with the YMCA. In 2019, an official memorandum of understanding was signed to signify long-term commitment to the partnership.
Develop educational materials	Developed a staff manual with details on the communication style (motivational interviewing), the program content (prediabetes, diabetes, diet and exercise), and additional supplementary information to support implementation (e.g., frequently asked questions section). Developed educational videos on program content, how-to videos and videos of senior research staff facilitating the program to clients to supplement the in-person training. All staff have ongoing access to videos on an online training platform.
Distribute educational materials	Each staff was provided a hard-copy program manual in addition to access to an online training platform with additional educational, how-to, and senior research staff facilitating the program videos.
Identify and prepare champions	Identified and trained one site lead per site to oversee and support staff, liaise with research team and support the program. Selected and trained a project coordinator to oversee sites and liaise with site leads and staff.
Identify early adopters	Training was conducted in three rounds. First-round staff were early adopters who conveyed their experiences to others at their organization and provided support to the subsequent round of staff.
Make training dynamic	In-person training was delivered with a variety of tasks such as PowerPoint slides, role-play, videos, discussion, and hands-on learning, practicing, and skill demonstration. In addition, ongoing access was provided to an online training platform with additional videos (shadowing, educational, and how-to videos).
Obtain and use patients/consumers and family feedback	All clients in the program were offered to participate in an optional interview at the end of the program. In addition, all clients were provided with surveys that collect program outcomes and program feedback at multiple time-points.
Promote adaptability	Staff were taught to deliver core program content (diet, exercise content, and exercise protocols) in a client-centered manner, providing flexibility on delivery. Key program content must be provided to each client but can be tailored to the client, e.g., order of content delivery, specific details. In addition, clients get a choice of exercise protocols (high-intensity interval training or moderate-intensity continuous training) and exercise mode (walking, cycling, elliptical).
Shadow other experts	Videos were created with a senior research staff facilitating the program to a client. As part of the mandatory training, all staff viewed segments of the videos demonstrating delivery of core program content. Full length videos were also added, and staff were encouraged to watch the full-length videos of a counseling session to understand session flow. As part of the mandatory training, all new staff had a senior research staff shadow them while they facilitated their first client through the program (expert shadows new staff).
Stage implementation scale up	During the planning process two local sites were selected for the first stage. Building on the success and lessons learned from the first sites, a third local site would launch. The project would continue to scale-up in a such a staged process, continually building on lessons learned.

Note: This table was compiled using the implementation strategies and definitions as described from the Expert Recommendations for Implementing Change (ERIC) project [18]

During the planning process, three local YMCA sites were considered. The first site had hosted SSBC since 2017, with research staff implementing the program. Despite exposure and prior knowledge of SSBC, staff were not involved in the program until the start of this current study. The second site was selected as the prospective staff were committed to the project and the organizational structure was similar to the first site. Due to differences in organizational structure and site readiness, the third site was selected to be a future scale-up site. This decision helped keep the project manageable and provided a location to implement lessons learned.

### Paradigmatic position

A pragmatic epistemology was used to guide the design of this research, which prioritizes using research findings to develop practical recommendations to answer the research question and tolerates multiple truths [29]. A qualitative descriptive methodology was used to guide interpretation of the qualitative data. This methodology compliments a pragmatic epistemology as qualitative description is useful when seeking practical answers through understanding a phenomenon from the perspective of those involved [30, 31].

### Participants and data collection

Each site nominated one staff as site-lead. This study involved two sets of participants: (a) staff who delivered SSBC and (b) implementation support staff. YMCA staff at both the delivery and implementation support staff level were invited to participate in the 1-year planning process. After hearing about the project, interested staff volunteered to enroll. Training occurred in two rounds and covered the counseling style, motivational interviewing (MI), and program delivery (see [32] for detailed description). Staff unable to attend the first training, in addition to any new staff wanting to enroll, were offered to attend the second training. Implementation support staff included the manager and site lead for each site, and the vice-president of health, fitness, and aquatics for the region. Ethical approval was obtained from the researchers’ institution, and written informed consent was obtained from all participants.

#### Semi-structured interviews

All YMCA staff (*n* = 8) who received training and counseled a minimum of three clients through the program were invited, and agreed, to participate in a semi-structured interview between 4- and 6-months post training (*M* = 5 months). This procedure was chosen to ensure staff had sufficient experience implementing the program, prior to an interview. Within the study period, the eight YMCA staff facilitated 32 clients through the program. The purpose of the interview was to understand the implementation strategies (e.g., training, support, program resources), provide feedback on the program, and understand the experience participating from an individual level. An interview guide was developed from previous research [33] and piloted with two staff, resulting in minor modifications (e.g., question wording, question addition; see Table 2 for sample interview guide questions). Staff ranged from 24 to 51 years old (*M* = 31.8, *SD* = 9.0), had between >1 and 17 years of experience at the community organization (*M* = 7.8, *SD* = 5.9), identified as 78% women and 89% Caucasian. Interview options (in-person [*n* = 8], or telephone [*n* = 1]), were provided to prioritize participant convenience and reduce burden. Staff interviews lasted 50–68 min (*M* = 58 min).

**Table 2:** Sample interview guide questions

	Interview questions
Staff interviews	Tell me about your overall experience as a trainer for SSBC. How has the SSBC been running at your YMCA so far? In what way has the program implementation met your expectations and/or needs? What challenges have you experienced? How do you believe you have been impacted by being a SSBC trainer? What recommendations do you have for adapting the current program? What do you like about the SSBC?
Focus group	Tell me about how the SSBC has been implemented at your site so far? Do you feel that your team has all the necessary support in place to run the program at the YMCA? How satisfied are you with the program at your site? What recommendations do you have for adapting the current program to better fit within the YMCA? What has been working well/not working well related to implementing the program? In what way have you noticed that the YMCA has been impacted by having the SSBC at your site? Now that we have been implementing the program for 6 months, what do you think could have been better from the beginning?

#### Focus group

An in-person semi-structured focus group was arranged 6 months into the project with implementation support staff. Each site lead and one site manager were trained to deliver the program and had direct experience in program facilitation. The purpose of the focus group was to understand the implementation strategies (e.g., training, support, program resources) and experiences participating from an organizational level. The focus group also discussed challenges identified in the project and solutions to promote sustainability. A semi-structured interview guide was created, guided by identified implementation determinants in the literature, including questions on feasibility, acceptability, culture, complexity, and sustainability [34]. Focus group participants ranged from 24 to 51 years old (*M* = 38.4, *SD* = 11.1), had between >1 and 17 years of experience at the community organization (*M* = 10.0, *SD* = 6.1), 80% identified as women, and 100% as Caucasian. The focus group lasted 118 min.

### Data analysis

The interviews and focus group were conducted by the first author, audio-recorded, and transcribed verbatim. A note-taker was present during the focus group and documented non-verbal cues and other observations that helped inform the analysis. Transcripts were read multiple times to promote familiarization with the data. A template approach [35] was used to thematically analyze transcripts and NVivo12 software [36] facilitated data organization. Data was coded using an inductive-deductive approach. After data familiarization, the first author independently coded all transcripts inductively and then met to discuss codes with the second and third authors. Inductive codes were discussed and revised (e.g., consolidated, adjusted name) as necessary to ensure accurate interpretation. To avoid overlooking data, first transcripts were coded inductively to ensure salient data was captured before deductively linking to CFIR constructs.

The CFIR technical assistance website [37] provided resources to facilitate data analysis including definitions of each construct within each domain. Rather than selecting which CFIR domains to examine, each inductive code was compared to all the CFIR construct definitions. By doing so salient CFIR constructs from relevant CFIR domains emerged from the data and any unrepresented CFIR constructs were excluded. The first author created an initial template that aligned the inductive codes with deductive CFIR constructs. The template included a section of inductive codes linked to deductive CFIR constructs that identified suggested modifications for sustainability. This section of the analysis helped identify modifications to inform future scale-up efforts. The template was reviewed with the second and third authors. Throughout the process, codes were examined for fit and consolidated, or separated, to better align with CFIR constructs. Definitions of CFIR constructs were constantly compared to inductive codes to ensure appropriate interpretation during the linkage step. The initial template was reviewed and discussed in multiple rounds of coding with the second and third authors, with the first author returning to the transcripts to ensure appropriate representation of the data. Discrepancies were discussed between all authors until consensus was reached. Relevant quotations were selected to accurately represent the CFIR construct and the participants’ lived experiences.

## Results

Several constructs from each CFIR domain were matched to inductive codes, and all five CFIR domains were represented within the data: (a) process, (b) intervention characteristics, (c) outer setting, (d) inner setting, and (e) individual characteristics. The CFIR’s process domain was difficult to link to the current qualitative findings as the interview guide focused more on participants’ reflections on the implementation plan execution and less on the planning process. Where possible, evidence supporting the process construct was provided in addition to a short descriptive overview of the planning process up to this project. Results are discussed according to each CFIR domain (heading) and CFIR construct (italicized). Quotes to support each construct are presented in Table 3.

**Table 3:** Overview of CFIR constructs linked to inductive themes

CFIR constructs	Themes	Example quotes
**Process domain**		
Plan	Planning process	*I also think just even the collaboration between two organizations. I think the amount of effort and time that goes into a collaboration to make it work from outside, from a place where you guys [research staff] are comfortable leading it, into a community-based place that has tons of stuff going on all the time. (C7)*
Engage	Involvement of stakeholders in the planning process	*I feel like it was very inclusive. We've been included in different [planning] meetings and included in the two-year celebration, and that inclusion just made it feel like we were a part of it, almost like we were a part of it from the beginning in just that inclusion. And that was really nice. (C4)*
Opinion leader	YMCA leadership	*I have been a part of the team from the beginning and really helping [principal investigator] bring this program to communities. So was part of bringing SSBC to the downtown Y[MCA]. And I’m now here to support my team and to ensure that we can continue to grow this program into our YMCA and potentially in the future grow this program into other Y[MCA]’s across the country. (VP)*
Formally appointed internal implementation leader	Site lead	*I am a SSBC coach as well as the site lead for [site 2]. (C6)*
	Implementation team	*I have been on the implementation team since we started SSBC and then I also lead the SSBC program. (C7)*
Champion	Partnership spearhead	*Since I started at the Y[MCA] in 2006, I worked under [champion]. She was my manager from the beginning. And her goal since then has been to help individuals and help them change their behavior and focus on the ready-to-be-fit population; so the group of people who are inactive, maybe at risk of a chronic disease. That has been her sole focus and has become my sole focus since I started here… I think this program just fits in perfectly with that strategy that the Y[MCA] has had since 2006. (C7)*
Execute	Fidelity evaluation	*I've never really been a part of a research program during the research phase… So just learning how to be very diligent with paperwork and completing it. (C7)*
Reflect	Implementation processes	*We’ve come up with different strategies of how to overcome different situations [during the site meetings]. We’re able to have a [research] update … so everyone gets the same amount of knowledge and has time to ask questions and get answers and everything. (C1)*
**Intervention characteristics**		
Relative advantage	Motivational interviewing training an asset for YMCA staff	*I think this is exactly what we have been looking for since I started here. You know about [other YMCA program name] and same kind of thing, but we didn't learn about motivational interviewing at all and I really do think that the motivational interviewing, that is the key piece that we were kind of missing in all of our other programs. (C7)*
	Program structure supports client habit formation	*The biggest [difference] is just how you exercise with them for the first bit. You help them with a guiding hand a bit more because you see them so often right away; it's easier to establish a relationship and actually have them commit to exercise a little bit easier. Compared to having one meeting telling them what they should do and then them just going off and doing it. (C9)*
	Building connections to clients	*I love it SSBC. I think this is my favorite thing that I do here [at the organization], honestly. It's kind of a nice break in my day. I love what SSBC brings to people.*” (C5). *I really like getting to know the participant. It's very rare that we would spend…this much one-on-one time with the same client for three weeks in a row. You really get to know them and really learn about them. It's not just the surface stuff…you really build a good relationship with these participants. I'm pretty sure if they keep coming to the Y[MCA], I'll stay connected with them for a long time…. I really enjoy that aspect. (C7)*
Relative (dis)advantage	Limited staff and client capacity of program	*Currently we only have full time staff doing it. (C6)* *I think scheduling-wise, how many clients would each staff be able to take on without feeling maybe burnout and still maintaining our other appointment types that we have as well. (C3)*
Adaptability	Program can be tailored to the client for benefits	*All the topics are great. I like that you have the flexibility. If you needed to, you could do the exercise ahead of time and then do counseling after... I like that you have the flexibility in the meetings to discuss a different topic if that's what they want to talk about that day. The fact that it's very client-centered and not black and white, I really think that’s important. (C7)*
Complexity	Effortful work	*It's not even more work as it is just-- what way to put it? It's not like you're doing more, it's just a little bit more taxing to do. I feel like you're putting more of yourself into it which you only have so much each time. (C9)*
Design quality and packaging	Professionally packaged from research team	*I'm always writing out checklists for people to make sure they check all the boxes, so it's just nice having it seem like a very professionally put together program. It just makes my life as a coach so much easier; I can just really focus on the participant and delivering the program and not worry about the logistical aspects of it. (C3)*
**Outer setting**		
Cosmopolitanism	Research partnership	*It's re-educating the population if you will, that we are kind of at the forefront of health and partnering with the university and into your health and the community that way. I think the word is getting out that people know that if they've got a health condition, this is a safe place to come. Preventing future health issues. Having a quality research-based program within the Y[MCA], it just helps us accomplish what we're trying to do. (C3)*
Peer pressure	Supports vision for a community, health-focused gym	*As part of the Y[MCA], we've sometimes maybe struggled with our identity and that people think we're just a rec[reation] center and that our staff maybe aren't trained or certified above just a fitness instructor that teaches one type of class. No, we have not only very certified and qualified staff but also training that is backed by research. (C3)*
**Inner setting**		
Structural characteristics	Staff turnover	*It has been going very well; in total, we have three and a half trainers. [laughter] [Staff name] is still technically a coach, but unfortunately, she’s moving away and then [another staff name], just due to scheduling, isn’t able to take participants all the time. (C1)*
Networks and communication	Community of practice among staff	*Or if they have questions about SSBC-- or if we're having troubles with a participant, we give each other ideas. We talk about it a lot. (C5)*
	Team meetings	*It's really nice to have that check-in. It feels like much more open communication. We get to hear what's going on the [research team] end, you get to hear our experiences, and it gives us all the opportunity to come together if there’s something that, say, we need a solution to, collaborate on that and see what we can find. Then it gives us, as trainers, an opportunity to get together and have that discussion as well. Sometimes we're having a bunch of one-on-one conversations. We don't have the opportunity to speak a group at the same time, so it does give that opportunity. (C2)*
Culture	Everyone should be a SSBC staff	*There should be no reason why somebody opts out [of this opportunity] from our group. (M1)* *The implementation totally fits in with the YMCA mission, vision—all that stuff—and our strategic direction that we've been heading for the last 15 years. (C7)*
Implementation climate		
Compatibility	Program is a good fit within YMCA	*The value is with the training and what we're able to provide in terms of coaching the participants, so value to [clients] in terms of changing their health and then value to us YMCA because we're keeping these members around, and they're staying active longer, and we're able to help them get healthier. (C3)* *I like what it represents. I like what the goal is to do. The goal is to have participant-led health intervention, lifestyle change. It follows all the YMCA values, which I value in my day-to-day and what I try to bring to coaching, what I try to bring all my clients. (C8)*
	YMCA staff well-suited to deliver program	*I almost don't factor in the exercise part because it's so second nature to me. So that works well, but it's supposed to work well, right? I would have no excuse for it not working well. (C2)* *I think it's one of the reasons why people work at the Y[MCA] is because we're all about building relationships and fostering this community. That’s probably the biggest thing for me – the relationships. (C7)*
	YMCA helps to set-up clients for long-term success	*Membership is number one. Being able to tell [clients] after SSBC that I'm still going to be here, you can still come and see me, you can send me an email. You're coming to this gym… you can get hold of me. Having it here-- the access to the gym, with the pool, the AquaFit, stuff like that. Being able to bring their families in. I think our demographic here is pretty friendly... pretty accepting. Having it in the gym makes it not as nerve wracking to go to a different gym. They can get comfortable here, working with someone, and then kind of go on their own. Knowing that we're still in the office and they'll know us if they have any questions. (C5)*
Learning climate	Teamwork	*We're a good team and everyone feels comfortable talking to anybody if they have a situation. (C6)* *The team is great. I have known some of them previously and sometimes if I get there-- because I go in early to get myself prepped—so if I'm having a challenge, I might say “I don't know what to do about this” and I might say, “I'm thinking this or that” and they're like, “Yeah. That sounds right. Go try it. Let us know how it goes”. (C3)*
Readiness for implementation		
Leadership engagement	YMCA managerial support	*I had that [check-in] talk with [staff name], but I know they can handle that content, it’s just helping them and pulling them of the cliff, right? I think it just comes down to us [managers] motivating them [staff], supporting them. (M1)*
	Site lead role	*It's really helping find their [staff] schedules and getting them booked in, helping them juggle things around if all of a sudden, we get a client that wants this timeslot, it's like okay, we can shift things. The new coaches that came on, we've had a couple touch-ins. The older coaches, the first-round coaches, not so much anymore. (C6)*
Available resources	Research team support	*Sometimes I'll have a question in my mind that I will want to ask one of you. Then all of a sudden, you're already here. It's easy to just ask, right? If I don't, I just email and usually within that day, I have a couple of questions answered. And if I have a follow-up to that, I'm pretty confident that it's going to be responded to that day as well. The support has been great. (C2)*
Access to knowledge and information	Implementation support tools	*I use my binder, the checklist, and I think those tools help me guide the session really well with knowing that it's okay to go in other topic directions if that's the way your client wants to go. (C7)* *I feel like I have all the resources and supports, the checklist and all of that. I feel very prepared to know what needs to be covered and it takes me maybe 5 to 10 minutes to go into the room, get all the equipment out that I need, the checklists, maybe flip through the coach manual. (C3)*
**Individual characteristics**		
Knowledge and beliefs	Belief that the program is impacting the clients	*I think it's been amazing. I love almost every single aspect about SSBC. I truly believe people are able to change after our meetings that we have with them. (C7)* *All three of my clients have admitted to me that they've taken something away from this program that has helped their life and has made them healthier. That’s a great feeling to have. This program has done so much for not only my three participants, but for multiple participants that have been coming back to the Y[MCA] and telling me how great this program is. (C1)*
Self-efficacy	Self-efficacy increases with more experience	*The more, I think, different people you interact with, the more confident you are. Some people are more confident, some meet all their goals and are super successful. Then some people don't. It's just navigating those different situations; there are always new situations. But once you've had experience with several, it's easier to think of what could be helpful to say next. (C3)* *It just goes back to the more you do it, the more confident you get. (C9)*
Individual identification with organization	Committed staff	*I look at it [program manual] before every session. I'm not going to go by my memory about what the session is supposed to be about. I read through the script and find that I just go over everything during that session with every client. (C6)* *Just like with any skill, it gets rusty, so that's why I really try to use it in all aspects of my personal and professional life when I first went through the training because I really was like, ‘I want to hone in on this’. (C3)*
Other personal attributes	Building transferable skills for personal and professional development	*I really like spending time in improving my communication skills because in my lifetime of doing that, the ability to communicate with someone better and avoid a crisis between a person or to understand them on a greater level and connect with them and feel connected, as well as understood, is really important. I feel like utilizing them in my personal or/and professional life outside of all of that is the best way to continue with them. It's not like I have to switch it on for a client and then switch it back off. It's always there to some degree.” (C4)* *The training I found very valuable. It's really made a difference with not just SSBC, but it's made a difference with other clients or other people that I'm working with too. The whole training experience and understanding motivational interviewing has been a very good experience. (C6)*
	Learning opportunity participating in SSBC	*The benefit is definitely the training and motivational interviewing; you can use that across the board with all clients any time. It’s further education for our trainers. It’s very impactful the way we speak to people and makes such a difference helping [clients] change. That is a huge benefit. (VP1)*

### Process

As noted, a 1-year *planning* process with the community partner *engaged* relevant stakeholders in co-developing the implementation *plan* [27]. A *champion* from the community organization spearheaded the partnership and was a respected, credible YMCA employee with formal influence on YMCA leadership, sufficient engagement in the initiative, and capacity to participate in the planning process. An *opinion leader* was invited to contribute to the planning process, a respected YMCA employee with formal influence as Vice-President of health, fitness, and aquatics. A product from the planning process was the creation of a *formally appointed internal implementation leader* (site lead), a role designed to facilitate communication between the research team and the site. A second product was the creation of a *formally appointed internal implementation team*, designed to have the champion, the opinion leader, and one front-line staff attend monthly implementation team meetings during the project. To appease both partners’ interest in assessing fidelity, an evaluation was developed to examine whether the YMCA staff *executed* the program as intended (see [32]). Finally, *reflecting and evaluating* was targeted both through formal evaluation (e.g., data collection tools) and ongoing reflection at monthly meetings at both implementation team and community organization staff levels. One CFIR construct in the process domain was unrepresented: *external change agents*.

### Intervention characteristics

Staff praised the *design quality and packaging* of the program, training, and associated documents (e.g., manual, checklists). Although there was specific program content to be delivered, each staff could deliver the session content in the order, manner, and depth that made sense to each client. This *adaptability*, encouraged by the client-centered nature of the program, was described as a facilitator. Staff were able to contextualize each appointment to their client. Although no staff described the program as difficult to deliver, staff did mention perceived *complexities*. A few staff discussed appointments being emotionally taxing from the effort needed to use and apply MI techniques. Even 3 months into program implementation, staff continued to prepare for each session. This, coupled with having some sessions run over-time, caused additional stress, especially when staff had appointments either right before or right after a scheduled SSBC appointment.

YMCA staff described the *relative advantage* of SSBC compared to other YMCA programs. Specifically, three different advantages emerged. First, participants described the MI training as an asset that should be used in other YMCA programs. MI emerged as a valuable skill transferable to other YMCA roles (e.g., group fitness, one-on-one training, conflict management). Second, YMCA staff described a SSBC appointment as different compared to how YMCA staff typically work with clients. SSBC allotted more time with a client while they exercised, which enabled staff to help clients learn about the equipment and physical sensations experienced in exercise. In addition, typical YMCA appointments did not include counseling time, which enabled staff to connect with their client, and genuinely listen and support them. Many staff were surprised by the amount of emotional support given to clients, requiring a tissue box in the counseling office. In addition, the counseling component allowed staff to discuss topics beyond traditional YMCA programs focused on physical activity, such as providing program-specific dietary content. Third, the staff felt the program structure (brief 3-week program, with home days) coupled with the behavior change techniques (e.g., action planning), helped clients build self-management and self-regulatory skills to build a diet and exercise habit. Staff explained that meeting a client regularly for 3 weeks is atypical for YMCA programming and enabled them to build close connections to clients. For many staff, they appreciated and enjoyed these differences between SSBC sessions and their other YMCA programming.

While the SSBC provided many perceived advantages relative to other YMCA programming, the program also included some *relative disadvantages*. Due to the schedule of SSBC sessions, only full-time staff could reasonably take-on clients as part-time staff could not accommodate the schedule needed in the first week of the program. In addition, many staff could only take on one client per month, which limited client capacity. Finally, while many of the CFIR constructs in the intervention characteristics domain were present, four were not: *intervention source*, *evidence strength and quality*, *trialability*, and *cost*.

### Outer setting

Staff discussed how SSBC helped with *peer pressure* by setting the YMCA apart from other fitness facilities. Providing SSBC further supported their niche as a community, health-focused fitness facility that provided accessible and effective programming to support the health of the community. In addition, the YMCA helps fill a gap in the local health authority, private, and public health through supporting a diabetes prevention program. The *cosmopolitanism* of the YMCA, through its partnership with a research group, supports this community, health-focused vision by offering an evidenced-based program. Two CFIR constructs were not represented in the outer setting domain: *patient needs and resources* and *external policy and incentives.*

### Inner setting

During the project, evidence emerged that *networks and communication* within both community sites supported staff with implementation. Staff utilized both formal communication during SSBC meetings and informal communication through a community of practice with other staff to discuss the program, troubleshoot, and practice skills. There were distinctions between *structural characteristics* within the two sites. One site was more established and larger compared to the other site (opened 2001 versus 2017, respectively). These differences impacted the social architecture, whereby the older site had a more “tight-knit” feel amongst staff compared to the newer site. The newer site also had more staff turnover compared to the older site.

Overall, the community organization had a supportive *implementation climate*. Staff saw SSBC as *compatible* with the YMCA at three different levels: (a) organization, (b) staff, and (c) client levels. First, SSBC was a good fit in the YMCA with its focus on helping individuals become healthier, especially in relation to increasing physical activity levels. Second, staff were well-suited to deliver SSBC as they shared many values with the program, including being passionate about health and fitness and helping individuals improve their overall health. Third, the YMCA provides an ideal environment for the clients to continue to achieve their goals post-program. While the program may end after the final session, the YMCA environment remains available for clients to benefit from. Relatedly, the organization had a positive *learning climate* where all participants worked together, regardless of rank in the organization, to implement the program and support each other.

The project was *ready for implementation* through its *leadership engagement* (site lead). The site leads supported implementation by helping with logistics (e.g., scheduling), supporting coworkers (e.g., check in with staff) and liaising with the research team. The project had *available resources* such as a dedicated project coordinator to provide ongoing support and lead monthly meetings. In addition, staff had *access to knowledge and information* through provision of implementation support documents (e.g., program manual, checklists) and ongoing access to an online training platform with support videos. Finally, discussions in the focus group identified a shared belief amongst management that everyone at the YMCA should be a SSBC staff, providing insight into the presumed *culture* at the YMCA to support such a program. While many CFIR constructs were represented within the inner setting domain, four constructs were unrepresented: *tension for change*, *relative priority*, *organizational incentives and rewards*, and *goals and feedback*.

### Individual characteristics

Staff’s *knowledge and beliefs* of the program grew during implementation. Staff discussed benefits of program tools, program structure, and counseling style (MI), and believed that the program positively impacted clients. Overtime, staff’s *self-efficacy* for implementing the program increased, particularly regarding use of MI. Evidence demonstrated *individual identification with organization* as staff displayed commitment to the success of the pilot project. Strong indications of commitment included staff completing preparation time (including preparing at home), reflecting on their skills, and practicing skills amongst each other. However, staff also mentioned being impacted by the program itself and described wanting to do well in their role to better help the clients. This code was placed within this domain (individual characteristic), as some staff demonstrated more commitment than others. However, the outer (*cosmopolitanism* of the research group with the YMCA of Okanagan), inner (*learning climate* and *culture*), intervention (*relative advantage*), and process (*engagement* in the planning process) likely contributed to staff’s strong commitment.

Staff identified *other personal attributes* from participating in the SSBC, such as building transferable skills for personal and professional development. Common skills described were listening, communication, teamwork, and conflict management. It was evident during the focus group that managers felt through providing SSBC, they were providing additional learning opportunities for their staff to build their resumé. One construct in the individual characteristics domain was unrepresented: *individual stage of change*.

### Modifications for increased sustainability

Ideas emerged during the focus group on how to modify the program for increased sustainability.

#### Intervention characteristics

To increase sustainability, the program has been restructured to reduce the time-burden on staff in the first week of the program, to a maximum of two appointments per client, per week. This modification eases staff perceived *complexity* by reducing the amount of time dedicated to SSBC per week. In addition, the restructuring reduces the identified *relative disadvantage* and allows part-time staff and volunteers to enroll in the training, resulting in an increased number of clients that the program can support each month.

#### Inner setting

To increase sustainability, SSBC team meetings have been incorporated into the larger YMCA site meetings to encourage all YMCA staff to become familiar with SSBC. This shift in the formal *networks and communication* within the organization aims to maintain all the benefits of the formal team meetings, but also encourages wider YMCA staff buy-in and eases scheduling as staff are already required to attend these meetings. This modification intends to further support the organizations’ *culture* identified in the focus group, to encourage more part-time and volunteers to sign-up for the training and make staff aware of the professional and personal development opportunities that SSBC provides to staff. The YMCA plans to continue to ensure *leadership engagement* through having managers and nominated site leads actively support staff who take the SSBC training through periodic check-ins. Some staff may need additional support and/or training to feel confident in implementing the program, which was indicated by the reliance on the research teams’ project coordinator (*available resources*). To encourage sustainability, the research team is working on additional training to transfer leadership from a research project coordinator to a coordinator within the organization. Training will support the individual assume a similar role to the project coordinator from the research team (e.g., lead SSBC meetings, provide ongoing support, check-in on staff).

## Discussion

To reduce the growing burden of T2D, more diabetes prevention programs need to be made available to those at-risk. To meet the need, there has been a focus on translating effective and scalable diabetes prevention programs into sustainable community settings. While there have been numerous diabetes prevention programs translated to date, there is a lack of research documenting the process of such translation efforts, the contextual factors impacting implementation and identifying the implementation strategies used to promote program uptake. The research team partnered with a local not-for-profit community organization to offer Small Steps for Big Changes with a goal of scaling-up to the national level of the organization. A co-developed implementation plan was used to translate the evidence-based program to two local YMCA sites. With context at the heart of implementation, an implementation determinant framework was used to analyze the implementation process and elucidate key contextual factors. The CFIR was a useful tool to frame the results. Moving from inductive to deductive analysis helped identify salient themes from participant perspectives prior to deductively linking to CFIR domains. This process enabled deep interpretation of the data as linkages were made to the CFIR framework and prominent CFIR constructs were generated from the data rather than preselected. As the analysis progressed, findings demonstrated an interaction between all levels of the CFIR contributing to successful implementation, similar to previous studies [38, 39]. The current study contributes to the field an in-depth during-implementation evaluation reflecting on the process of implementing a program using the CFIR framework to guide analysis and identify modifications to inform future scale-up efforts. Such a study also provides an overview of implementation strategies used according to definitions described from the Expert Recommendations for Implementing Change (ERIC) project [28]. The analysis included multiple, in-depth perspectives from those involved in the project (front-line staff, managers, site-leads, vice-president of health, fitness, aquatics). All five CFIR domains were identified, with relevant constructs supported within each domain.

Overall, the implementation plan was successful, with positive views from all study participants. Results suggest the high level of engagement during the planning process with the community partner positively influenced the successful execution of the implementation plan. While a partnered approach takes time and effort, the process of building a partnership embeds commitment and critical thinking throughout, ensuring appropriate steps are taken and a quality product results [40, 41]. The current study supports literature that sufficient planning through early engagement, the inclusion of a wide range of stakeholders, and prioritizing planning for scale-up from the outset is essential for success [42, 43]. A successful implementation context likely formed through strong commitment to the partnership at the leadership level of the YMCA (*opinion leader* and *champion*) and engagement of prospective SSBC staff at the YMCA, both important factors for implementation planning [38, 44]. Involvement of prospective staff developing the implementation plan, an often-overlooked part of implementation, likely fostered buy-in and encouraged adoption [12]. Engagement with the community partner ensured implementation strategies (e.g., provision of a staff training manual, program documents, ongoing implementation support through monthly site-meetings) were deemed acceptable and sufficient to front-line staff prior to implementation. Overall, the highly engaged, multi-level planning approach supported multi-level buy-in.

The YMCA has been identified as a promising partner to disseminate diabetes prevention programs [45]. Synergies were evident at multiple levels. The program itself was well suited to the vision of the YMCA being a community-health focused fitness facility [41] and the YMCA staff were well suited to deliver the program as they already had knowledge, skills, and desire to help clients within the health and fitness industry [44]. This synergy may have led to highly engaged staff, a facilitating factor for successful implementation [16, 43]. Participation in the project provided staff with new professional and personal development skills. Specifically, MI emerged as a valuable skill and a key component in the program. Staff discussed many relative advantages of SSBC, largely due to MI coupled with other program components (e.g., counseling component, diet and exercise focus, and 1-month program) that are not typically included in YMCA programming. Having relative advantage is another facilitator identified in prior implementation studies [46, 47]. Specifically, in a program for individuals with overweight and obesity, health care workers who perceived relative advantage of an intervention had higher motivation to implement it [48]. To further increase the advantages and support sustainability in the future, feedback from the current results on complexity and the relative disadvantage have been modified to support future scale-up. A new program structure will reduce staff burden during the first week, an allow greater flexibility in scheduling, and allow part-time and volunteer staff to feasibly fit appointments into their schedules.

All participants commented on the level of support provided with the program. Support was provided through multiple communication channels, both formal (e.g., team meetings, implementation team meetings, one-on-one support through project coordinator, site lead, program manual, checklists) and informal (e.g., community of practice, research team visit to sites). Multi-directional communication channels have been identified as a facilitator in previous research [44]. Specifically, health care professionals that perceived more support from their colleagues, had greater motivation to implement an intervention [46]. Relatedly, differences between the two sites emerged in the inner setting, with one site discussing more informal communication (e.g., seeking support from co-workers) compared to the other site. This difference may be attributed to the structural characteristics of the two sites with the “tight knit” site being in existence longer, more staff working at one time (enabling overlap and communication opportunities), a site lead at a higher organizational leadership level, and less staff turnover. These differences may have impacted the level of informal support provided at the younger site, as one staff at the younger site struggled with the intervention at the early stages, despite the amount of formal support. A timely change in site-lead, enabled the staff member to receive additional peer-support to gain confidence in implementing the intervention. In prior research, specifically in relation to a study using MI, providing resources and communication between colleagues has been shown to enhance facilitators’ ability and experience of delivering MI by having constant access to feedback and support [49]. These observations demonstrate the importance of selecting an appropriate site lead and the impact of the inner setting on social knowledge; both have been identified in research using the CFIR [50].

More research is needed to understand important factors to cultivate an inner setting conducive to adopting an intervention and executing the intervention with success. The current findings support the strategy of having a site lead; however, selection of an appropriate site lead and provision of training will likely enhance the strategies’ success. In a study investigating the effects of champions as an implementation strategy, results determined the success of the strategy was dependent on the chosen champion and adequate organizational support [51]. Four characteristics of a successful champion were identified: engagement, influence, credibility, and capacity [51]. Building off this study, coupled with the characteristics identified for a successful champion, future research is needed to operationalize the site lead implementation strategy.

It is important to note the amount of researcher engagement within the current implementation effort. The first author acted as project coordinator and was engaged in the planning of the pilot, development of implementation tools (e.g., training manual, training plan), oversaw the execution of implementation activities (e.g., attended and co-led staff training, liaised with site leads, led monthly site meetings, attended monthly implementation team meetings, provided ongoing support to front-line staff), developed close connections to participants in the project, and acted as a knowledge broker to pass information between the implementation team, research team, and the front-line staff. Close connections, open communication, and provision of support were likely facilitated by the size of the organization and the ability to have face-to-face meetings—two factors that have been identified as facilitators to successful partnerships in community health promotion [30, 44]. Feedback from a 15-year scale-up project identified that a local project coordinator was fundamentally important to implementation success [52]. Within the Kerala Diabetes Prevention Program, researchers had close communication with peer leaders (e.g., provided ongoing support via telephone calls prior to and after each group session) and utilized a local resource person (a leader nominated from the local self-governing bodies) to facilitate implementation (e.g., organizes group sessions, advocate for the program and send reminders/follow-up with participants) [14]. The engagement of peer leaders and local resource persons were crucial to implementation [14]. In the current project, the project coordinator was the researcher (first author) who ensured implementation strategies were executed and feedback collected. As the project continues to be scaled-up, the role of the project coordinator will shift to the YMCA site lead role. Careful selection and support to the site lead will be of utmost importance for successful implementation.

### Limitations and future directions

The first author participated in all front-line staff meetings, implementation team meetings and was involved in the 1-year planning process. This involvement enabled the researcher to observe and simultaneously support the implementation strategies throughout the project. Although this dual role may introduce bias, it has been used in similar implementation studies for an in-depth perspective of the implementation process [43, 53]. All participants regularly interacted with/collaborated with the first author. While these professional relationships likely resulted from the collaborative partnership approach, it can also introduce social desirability bias. However, the close working relationship also enabled mutual respect enabling participants to provide honest feedback to the research team. The first staff to volunteer for the project may have been more motivated compared to staff trained in subsequent rounds. The study was interrupted by COVID-19 and not all staff had facilitated three clients through the program prior to facility shut down/interruption, which reduced the study’s sample size. The sample was primarily Caucasian women, which may limit generalizability to other studies or new sites with a more diverse staff. Finally, the study design itself (e.g., interviews and focus group) were effective strategies to *reflect* on the process of implementation. Useful feedback was obtained to modify the program for increased sustainability with the community organization. Future research should include qualitative evaluation during implementation to understand the process and learn from it, to be able to make improvements in the future.

Two of four recommendations identified in a 2016 systematic review on the application of the framework [17] were applied in this study (meaningfully use the CFIR to inform future scale-up initiatives and to describe how CFIR constructs were selected). Future research should work to include the other recommendations: associating CFIR constructs with study outcomes and integrating the CFIR throughout the research process. While the CFIR was integrated throughout much of the research process, future work should consider aligning data collection materials (e.g., interview guides) with resources available on the CFIR technical assistance website [37]. In this present research, had the interview guide been developed to align with CFIR constructs, unrepresented CFIR constructs might have had representation. For example, all participants were aware that the intervention was developed externally (i.e., *intervention source*), had demonstrated effectiveness (i.e., *evidence strength and quality*) and was being piloted (i.e., *trialability*). However, by not aligning the interview guide with CFIR constructs, salient themes emerged inductively rather than asking participants about all constructs that may or may not be deemed relevant by the participants spontaneously. While the research team was not able to accommodate all recommendations from the systematic review, results contribute to the CFIR’s research base by identifying relevant constructs within this project’s successful implementation effort.

The implementation strategies used in the project support their use in future scale-up efforts, which are underway. The current study was not able to assess which specific implementation strategies were most effective, rather the results presented are a result of the package of strategies used. Future experimental research should be used to tease apart the effects of specific implementation strategies and/or future systematic reviews and meta-analysis can be used to identify promising implementation strategies. In the current project, co-developed implementation strategies were executed with researcher support. In the next phase of program scale-up, implementation strategies will be executed by community organization staff. This tiered approach has been successful in prior research [53], after building off an initial study with strong involvement of the researcher [43, 52, 53]. The two sites where the project was conducted are well-suited to take on such leadership responsibilities having closely collaborated and shaped the current form of the program and will be invited to share lessons learned with new scale-up sites. When setting up a new site, sufficient support and engagement with prospective staff is warranted. While engagement requires additional resources and time, it has favorable outcomes when compared to more hands-off approaches (e.g., provision of a manual) that may not be sufficient to support implementation [38, 52]. Future research is needed to optimally operationalize provision of support in a sustainable way.

## Conclusions

This study provides an in-depth analysis into the process of executing a co-developed implementation plan to have staff facilitate an evidence-based diabetes prevention program within a community organization. Overall, the implementation strategies used were acceptable and staff were satisfied with the implementation process. Project success was likely a result of the process in which they were developed, planned, executed, and reflected, which provides valuable feedback to prepare for the future scale-up of the project. This study demonstrates an effective partnered approach to the process of implementation and demonstrated important constructs from all five levels of the CFIR.

## Supplementary Information

**Additional file 1: Supplementary file A.** The TIDieR (Template for Intervention Description and Replication) Checklist. Information to include when describing an intervention and the location of the information.

## Data Availability

The datasets used and/or analyzed during the current study are available from the corresponding author on reasonable request.

## Abbreviations

CFIR: Consolidated Framework for Implementation Research
MI: Motivational interviewing
SSBC: Small Steps for Big Changes
T2D: Type 2 diabetes

## Footnotes

1 The YMCA waived their rights to anonymity
