# Supplementary material for: Implementation of a diabetes prevention program within two community sites: a qualitative assessment
Source: Implement Sci Commun. 2022 Feb 5;3:11. doi: 10.1186/s43058-022-00258-6 (PMC8817168; doi:10.1186/s43058-022-00258-6)
Supplement: Supplementary file 1 — Additional file 1: Supplementary file A. The TIDieR (Template for Intervention Description and Replication) Checklist. Information to include when describing an intervention and the location of the information. [file 43058_2022_258_MOESM1_ESM.docx]

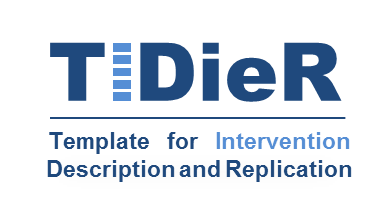
Supplementary file **A**

**The TIDieR (Template for Intervention Description and Replication) Checklist**

Information to include when describing an intervention and the location of the information

| **Item number** | **Item** |  |
| --- | --- | --- |
|  |  |  |
|  | **BRIEF NAME** |  |
| **1.** | Provide the name or a phrase that describes the intervention. | Small Steps for Big Changes Diabetes Prevention Program |
|  | **WHY** |  |
| **2.** | Describe any rationale, theory, or goal of the elements essential to the intervention. | Small Steps for Big Changes (SSBC) was developed using social cognitive theory and aims to foster long-term dietary and physical activity adherence through use of motivational interviewing (MI)-informed behavioural counselling sessions, supervised exercise sessions, and the application of evidence-based behaviour change techniques (BCTs). |
|  | **WHAT** |  |
| **3.** | Materials: Describe any physical or informational materials used in the intervention, including those provided to participants or used in intervention delivery or in training of intervention providers. Provide information on where the materials can be accessed (e.g., online appendix, URL). | The SSBC takes the form of a conversation about physical activity and dietary behaviour change at a local fitness facility. Clients are provided with a free one-month gym membership. Clients are asked to download a mobile phone application to self-monitor their diet and exercise. Additionally, clients are provided with a program workbook with brief informational resources related to program content, and space to take notes on the content discussed and their goals. The overarching purpose of this study was to describe the BCTs and MI techniques (MITs). These techniques are reported in the results section of the article. |
| **4.** | Procedures: Describe each of the procedures, activities, and/or processes used in the intervention, including any enabling or support activities. | The SSBC consists of 6 individual behavioural counselling and supervised exercise sessions over the span of ~3 weeks. Generally, the first session is directed at a program overview and exploration of client’s goals and motivation. The remaining sessions target exercise or dietary changes shown to decrease risk of developing type 2 diabetes (T2D; e.g., reducing sugar intake, increasing fruit and vegetable consumption, etc.). All sessions allow for diet and exercise goal setting and action planning if the client is ready to do so. Following the counselling sessions clients engage in either moderate-intensity continuous training or high-intensity interval training. In the first two sessions clients try out both types of exercise, in all remaining sessions they are provided with the choice of exercise type. Every session, clients are provided the choice between aerobic exercise equipment (e.g., treadmill, elliptical, stationary bicycle). Exercise sessions increase in length from 20 – 30 minutes for moderate exercise and 12 – 15 minutes for interval exercise. SSBC counselling and supervised exercise sessions include the delivery of BCTs and MITs.  Clients are encouraged to join our on-going community-engaged program initiatives which include a weekly participant-led walking group, monthly meeting held at the fitness facility, Facebook group and a quarterly program newsletter. Anyone is invited to attend these community-engaged program initiatives, even if they were not a part of SSBC. |
|  | **WHO PROVIDED** |  |
| **5.** | For each category of intervention provider (e.g., psychologist, nursing assistant), describe their expertise, background and any specific training given. | All sessions are one-on-one, take place at the fitness facility, and are conducted by a coach who has taken part in a 3-day training covering MI and program delivery. Coaches are fitness facility staff members. |
|  | **HOW** |  |
| **6.** | Describe the modes of delivery (e.g., face-to-face or by some other mechanism, such as internet or telephone) of the intervention and whether it was provided individually or in a group. | SSBC sessions are one-on-one and are delivered face-to-face. |
|  | **WHERE** |  |
| **7.** | Describe the type(s) of location(s) where the intervention occurred, including any necessary infrastructure or relevant features. | SSBC counselling sessions are delivered in a private counselling room within the two fitness facility sites and the exercise sessions are delivered in the YMCA gymnasium using aerobic equipment (e.g., treadmill, elliptical, stationary bicycle) located within Kelowna, British Columbia. |
|  | **WHEN and HOW MUCH** |  |
| **8.** | Describe the number of times the intervention was delivered and over what period of time including the number of sessions, their schedule, and their duration, intensity or dose. | SSBC counselling sessions typically last between 30 and 50 minutes, exercise sessions last between 12 and 30 minutes. Clients participate in 6 sessions over the course of ~3 weeks with a 1-month follow-up to check in on program progress. Sessions are scheduled to support self-management post-program and therefore clients engage in 3 sessions in the first week, two sessions in the second week and one final session in the third week of the program. Home-days are scheduled between each in-person session to enhance self-regulatory skills. |
|  | **TAILORING** |  |
| **9.** | If the intervention was planned to be personalised, titrated or adapted, then describe what, why, when, and how. | SSBC counselling sessions are tailored to the client’s readiness to change, interests, motivations, goals, schedule, etc. In addition, each participant is provided with a tailored heart rate range for moderate and high intensity activity. The target heart rate range is based on their baseline resting heart rate and provides a target for clients to achieve during the exercise sessions. |
|  | **MODIFICATIONS** |  |
| **10.^ǂ^** | If the intervention was modified during the course of the study, describe the changes (what, why, when, and how). | A modification to the program structure was introduced. The modification changes the session arrangement and length of the program to four weeks: two sessions in the first week, two session in the second week, and one session in week three and 4 four. |
|  | **HOW WELL** |  |
| **11.** | Planned: If intervention adherence or fidelity was assessed, describe how and by whom, and if any strategies were used to maintain or improve fidelity, describe them. | Intervention adherence is monitored through program checklists completed post-session by program coaches. The checklists represent key program topics, protocols and components to be delivered. In addition, each session is audio-recorded for MI-fidelity. Currently, monthly meetings occur with program staff to discuss questions, and program updates, where program fidelity might be discussed. |
| **12.^ǂ^** | Actual: If intervention adherence or fidelity was assessed, describe the extent to which the intervention was delivered as planned. | Fidelity was assessed, see [22] for results. Overall fidelity was high. |

** **Authors** - use N/A if an item is not applicable for the intervention being described. **Reviewers** – use ‘?’ if information about the element is not reported/not   sufficiently reported.

† If the information is not provided in the primary paper, give details of where this information is available. This may include locations such as a published protocol      or other published papers (provide citation details) or a website (provide the URL).

ǂ If completing the TIDieR checklist for a protocol, these items are not relevant to the protocol and cannot be described until the study is complete.
* We strongly recommend using this checklist in conjunction with the TIDieR guide (see *BMJ* 2014;348:g1687) which contains an explanation and elaboration for each item.

* The focus of TIDieR is on reporting details of the intervention elements (and where relevant, comparison elements) of a study. Other elements and methodological features of studies are covered by other reporting statements and checklists and have not been duplicated as part of the TIDieR checklist. When a **randomised trial** is being reported, the TIDieR checklist should be used in conjunction with the CONSORT statement (see [www.consort-statement.org](http://www.consort-statement.org)) as an extension of **Item 5 of the CONSORT 2010 Statement.** When a **clinical trial** **protocol** is being reported, the TIDieR checklist should be used in conjunction with the SPIRIT statement as an extension of **Item 11 of the SPIRIT 2013 Statement** (see [www.spirit-statement.org](http://www.spirit-statement.org)). For alternate study designs, TIDieR can be used in conjunction with the appropriate checklist for that study design (see [www.equator-network.org](http://www.equator-network.org)).
